# To assess whether addition of pyriproxyfen to long-lasting insecticidal mosquito nets increases their durability compared to standard long-lasting insecticidal mosquito nets: study protocol for a randomised controlled trial

**DOI:** 10.1186/s13063-015-0700-7

**Published:** 2015-04-28

**Authors:** N’Fale Sagnon, Margaret Pinder, Emile FS Tchicaya, Alfred B Tiono, Brian Faragher, Hilary Ranson, Steve W Lindsay

**Affiliations:** Centre National de Recherche et de Formation sur le Paludisme (CNRFP), Ouagadougou, Burkina Faso; School of Biological Sciences and Biomedicine, Durham University, Durham, UK; Medical Research Council Unit The Gambia, PO Box 273, Banjul, The Gambia; Centre Suisse de Recherches Scientifiques en Côte d’Ivoire, Abidjan, Côte d’Ivoire; Liverpool School of Tropical Medicine, Liverpool, UK

**Keywords:** Net durability, Insecticide resistance management, Malaria control, Insecticide-treated bed net, Pyrethroid, Pyriproxyfen, Insect juvenile hormone mimic, Clinical malaria, Entomological inoculation rate, Randomised controlled trial

## Abstract

**Background:**

The effectiveness of pyrethroid-treated bednets for malaria control in sub-Saharan Africa is under threat because of high levels of resistance to pyrethroid insecticides in the vectors. Here we assess the durability of polyethylene nets with a novel combination of permethrin, a pyrethroid, with pyriproxyfen, an insect juvenile mimic (PPF-LLIN), in comparison with a typical permethrin-treated long-lasting insecticidal net (LLIN).

**Methods:**

This is a cluster randomised controlled trial of net durability in Burkina Faso, with clustering at the level of the compound and includes entomological outcome measurements. Half the compounds in each village will be randomly allocated PPF-LLIN and half the LLIN. All sleeping places in a compound will be provided with one type of net. We will distribute the nets at the start of the first transmission season and follow net use at the start and end of each transmission season for 3 years. In one village, bio-efficacy and chemical content will be recorded immediately after net distribution and then at 6, 12, 18, 24, 30 and 36 months. In the other village net survivorship and fabric integrity will be recorded immediately after distribution, and then at 6, 12, 18, 24, 30 and 36 months. Routine measurements of indoor temperature and relative humidity will be made in both villages during the study. Residents will be followed for possible side effects of the PPF-LLIN by surveillance of known asthmatic subjects during the first month post-distribution and pregnancy outcomes will be monitored from antenatal clinic records.

**Discussion:**

The protocol is novel on two accounts. Firstly, it is the first to describe the procedure for measuring net durability following recent World Health Organisation (WHO) guidelines. Meeting the minimum requirements set in the guidelines is essential before a new type of net can be recommended by WHO’s Pesticide Evaluation Scheme (WHOPES). Secondly, it describes methods to monitor the persistence of an active ingredient that reduces vector fertility and fecundity. If the PPF-LLIN is both effective and persistent it will provide an alternative vector control strategy where pyrethroid-resistant vectors are present.

**Trial registration:**

ISRCTN30634670 assigned 13 August 2014.

**Electronic supplementary material:**

The online version of this article (doi:10.1186/s13063-015-0700-7) contains supplementary material, which is available to authorized users.

## Background

The massive scale-up of long-lasting insecticidal nets (LLINs) has contributed to a decline in malaria in many parts of sub-Saharan Africa (SSA) over the past decade [1,2]. Pyrethroids remain, until now, the only class of insecticide used for treating nets since they are highly insecticidal and safe to use [3]. However, the rapid increase in resistance to pyrethroid insecticides in mosquitoes may jeopardise future malaria vector control [4]. Polyethylene nets, containing a novel combination of permethrin, a pyrethroid, and pyriproxyfen (PPF-LLIN), an insect juvenile hormone mimic [5-8], may enhance malaria control, as well as reducing the spread of pyrethroid-resistant mosquitoes.

A two-armed cluster-randomised controlled trial (AvecNet) [9] is being conducted near Banfora, Burkina Faso, to assess whether PPF-LLINs (Olyset Duo nets containing 2% permethrin, 1% pyriproxyfen w/w) provide better protection against clinical malaria in children than 2% permethrin-treated LLINs (Olyset Nets) in an area with high levels of pyrethroid resistance in the main vector, *Anopheles gambiae s.s*. [10]. At the start of the study, 33% mortality was obtained after exposure to a discriminating dose of permethrin (0.75% papers for 1 hour exposure) and the time to kill 50% after exposure to the discriminating dose was 91 minutes. In the current study we propose to measure the durability of both PPF-LLINs (Olyset Duo) and LLINs (Olyset), used as a control, under operational conditions for 3 years to complement the efficacy results of the main trial. Since all villages in the main trial will have PPF-LLINs by the end of the 2-year trial, this durability study will take place in villages outside the main trial and adverse events (AE) will be monitored as for the main trial.

Recently it has become apparent that while the Olyset brand of LLINs has excellent tensile strength and insecticidal properties that last for many years, the netting is prone to develop elongated holes due to the weave unravelling once a small hole develops. Thus, large-scale surveys, supported by the President’s Malaria Initiative, of national LLIN distributions in several African countries found that after 2 years of domestic use, Olyset nets sometimes scored badly on fabric integrity in comparison to many other brands [11]. The manufacturer has acted on these findings and changed the knitting pattern of the Olyset Net, which inhibits the development of elongated holes, so that since late 2013 all Olyset Nets manufactured have this new knitting pattern (J Lucas, personal communication). In our durability trial we will use new knitting pattern nets for both the PPF-LLIN and LLIN. Durability of PPF-LLINs and LLINs will be assessed using 3 criteria: (1) net survivorship, (2) fabric integrity and (3) insecticidal activity (bio-efficacy). This protocol follows the recommendations made by WHO in 2013 entitled ‘*Guidelines for monitoring the durability of long-lasting insecticidal mosquito nets under operational conditions*’ [12], WHO’s 2013 ‘*Guidelines for laboratory and field testing of long lasting insecticidal nets*’ [13] and WHO’s Vector Control Technical Expert Group’s guidelines on ‘*Estimating functional survival of long-lasting insecticidal nets from field data*’ [14].

This protocol is the first we know of that adheres to these guidelines. It therefore serves as an exemplar for other studies wishing to adopt these criteria.

### Study objectives

#### Primary objective

To determine whether the bio-efficacy of PPF-LLINs is superior to that of LLINs for the first 36 months after net distribution.

#### Secondary objective

To determine the physical integrity of LLIN and PPF-LLINs for the first 36 months after net distribution.

#### Tertiary objectives

1. To determine the survivorship of LLIN and PPF-LLINs for the first 36 months after net distribution.
2. To measure the content of permethrin in LLINs and permethrin and PPF in PPF-LLIN netting for the first 36 months after net distribution.
3. To document AE that may be associated with PPF-LLIN use.

## Methods/Design

### Study area

The study will be carried out in two villages in the Cascades Region of Burkina Faso. In Dalamba (village A) samples will be cut from nets and the nets will be replaced (Primary objective and Tertiary Objective 2) and Sanako (village B), where nets will be left intact (Secondary objective and Tertiary Objectives 1 and 3). AE will be followed in both villages. The separation of activities in two villages is designed for operational reasons to simplify data collection and analysis since in village A nets will be replaced through the course of the trial, whilst in village B nets will be delivered and hung only at the start of the trial. This arrangement is also less likely to create dissatisfaction amongst those who will not get replacement nets. The study villages have a total population of approximately 2,000 and are situated close to the south-east of Banfora (10° 56′ 00″ N, 004° 46′ 00″ W) in an area of open Sudanian savannah. These villages are located near those enrolled in the main AvecNet Trial, but are outside the main study area. Most people in the main trial and in this study live in houses made with mud or cement walls and thatched or metal roofs. The climate is tropical with most rain falling from May to October. This defines the highly seasonal malaria transmission with most malarial episodes experienced during or immediately following the major rains. The study’s field station is based at Banfora town, which is the main town in the district. This is an area of cotton-growing since 1968 with *An. gambiae* S forms predominating over M forms [15].

### Net owner enrolment

As is customary in Burkina Faso, sensitisation will start by discussions with community elders and then representatives of the villages in order to explain the nature of the study and what will be required during the study (information sheet, Additional file 1). The key attendees names and roles will be documented for each village.

We will explain to individual net owners the purpose of the study and will provide them with an information sheet (Additional file 1). Witnessed written consent (Additional file 1) will be obtained from each participant, before net donation and exchange of their previous net. We will also seek written consent from net owners before taking netting samples. During these procedures it will be made clear that subjects and households will be able to leave the study at any time without giving a reason but their original net will not be returned. Given the nature of the study we will also encourage participants to keep the nets they are given and not to exchange them. We envisage that most study subjects will be enrolled at the start of the study for 36 months. Occasionally, due to movement of residents into the villages, some subjects join during the study.

### Randomisation and blinding

Both types of net will be randomly allocated to compounds, with allocation stratified by compound size in both villages. In village A 6 different compounds will be sampled in each study arm at 7 sequential time points: thus, 84 compounds (42 for each study arm) will be selected randomly, without replacement, at the start of the study. The remaining compounds in the village will be randomly assigned equally to the intervention or control and will act as reserve compounds in case of withdrawal of those randomised. At each sampling time point 4 nets will be randomly selected from each of the pre-selected compounds: that is within 1 to 2 weeks of hanging and then 6, 12, 18, 24, 30 and 36 months later. As holes will be cut in the selected nets to provide netting samples for laboratory testing, the nets will be taken from the recipient and replaced with a new one of the same type: that is LLINs will be replaced with LLINs and PPF-LLINs replaced with PPF-LLINs. A total of 649 nets will be distributed in Dalamba (village A) and 541 in Sanako (village B). In cluster-randomised controlled trials it is particularly important to minimise imbalance for factors likely to be correlated with the outcome; bio-efficacy will probably be affected by net washing and possibly by the ambient temperature and humidity, this will be corrected for in the analysis. Observer bias will be reduced where feasible. Both types of nets will be of a similar shape and colour and will be blinded before distribution, see below. Thus, end-users, field workers conducting the physical integrity/survivorship surveys and technicians conducting the bio-efficacy, fabric integrity and insecticide content tests will be blinded to the type of net. A standard questionnaire will be used to collect data from the net user at each follow-up. The processes of the interventions, both for PPF-LLINS and LLINs, will be closely monitored not only for quality but also to document any bias between the clusters and villages.

### Interventions

The LLINs and PPF-LLINs distributed will be manufactured by Sumitomo Chemical Company (Japan) and are the same as those being used in the main AvecNet Trial, model ‘Extra Family’ rectangular (180 cm wide × 190 cm long × 150 cm high), except that these will have been manufactured using the improved (new) knitting pattern. The LLINs are WHO recommended and meet WHO specifications (http://www.who.int/whopes/Long_lasting_insecticidal_nets_Aug09.pdf) with 2% w/w permethrin incorporated into polyethylene fibres. The PPF-LLINs contain 2% w/w permethrin and 1% w/w pyriproxyfen incorporated into polyethylene fibres. Olyset Duo was submitted to WHOPES in August 2012 and has recently completed Phase I testing. These nets will be distributed at the beginning of the main transmission season, at no cost to the recipients. Government Roll Back Malaria information, education and communication procedures will be followed to encourage correct net use and maintenance. The storage and distribution of the LLIN and PPF-LLIN will follow the same procedures as detailed in the main AvecNet Trial Protocol (submitted June 2014). In addition, as this trial will be blinded for the recipients and survey field assistants and laboratory staff, nets will be given sequential unique identity (ID) numbers in the top right hand corner of the label and all mention of ‘Olyset’ and ‘Olyset Duo’ removed before the start of net distribution. This will include blinding or replacing bags in which the nets are supplied from the manufacturers. The ID and net type will be noted on specific forms, which will kept in a sealed envelope and locked under the supervision of the Principal Investigator (PI) until the datasets are finalised and locked.

The date the nets are given to the householder will be written under the unique identifier at the time of donation so that all nets will be numbered with a unique ID number and the date of issue. The recipient’s name, net number, date of donation and identity of the compound and household to which they were distributed will be recorded on the clinical report form (CRF) at the time of delivery. The household identification, name of head of household, and global positioning system (GPS) coordinates will be recorded at the baseline demographic survey. Both types of net will be the same design and colour so that study participants will be blinded to the net allocation. However, each net ID will be coded and the location and recipient recorded as indicated above and this information will be collected at each survey to keep track of the identity of each net.

### Study procedures and evaluations

The study villages will be enumerated following normal demographic surveillance procedures and compounds mapped before being randomised to net type. It is intended that LLIN distribution will be completed by July/August 2014. Nets selected for monitoring will be identified by their unique ID, date of donation and located by the village, household number, name of the head of household and user name. Net users who have moved, and taken their nets with them, or withdrawn consent will be recorded on the survey form and on the master list and they will be replaced by another net user from the same compound. If an entire compound refuses to participate another compound will be randomly selected from those remaining.

Surveys will be conducted in village B at 0, 6, 12, 18, 24, 30 and 36 months post-distribution for all endpoints. A standard questionnaire will be used to collect data at each follow-up from the net user, or parent/carer in the case of children. The following information will be recorded: whether the net is hung over the sleeping place at the time of visit, whether it is in storage, being washed, given away or discarded, the method of hanging, whether it can be tucked securely under the mattress all around, and the fabric integrity and the condition of the net. In village B all available nets will be surveyed each time point, in village A only the compounds randomised for sampling at each time point will be surveyed.

### Net selection and sampling

**Figure 1:**
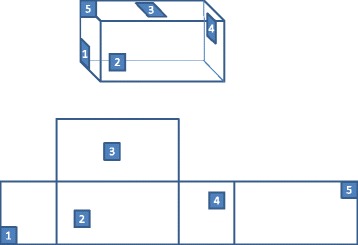
Position of samples taken from each panel of the bednets [12].

Village A will be used to sample nets for bio-efficacy assay and the measurement of insecticide content, as detailed in ‘randomisation and blinding’ above. Samples will be cut from the nets while they are hanging in the villages. The bio-efficacy assay requires 3 samples of 25 cm^2^ from each net (Positions 2, 3 and 4; see Figure 1). Following the WHO guidelines [10,11] the measurement of insecticide content will made on 5 pieces of 30 cm^2^ from each net immediately after distribution (positions 1 to 5 in Figure 1) and 4 pieces of 30 cm^2^ from each net 6, 12, 18, 24, 30 and 36 months later (positions 2, 3, 4 and 5). The bio-efficacy samples will be wrapped individually in aluminium foil but all the samples taken to measure insecticide content from a single net will be wrapped together. After wrapping in foil the samples will be labelled with the type of assay, the date of sampling and the net ID plus the position sampled (for example, 092–3 is net ID 92, cut from position 3). Samples will be stored at +4°C, and left for 24 hours at room temperature until assayed.

Village B will be used to measure net survivorship and fabric integrity immediately after net distribution and then 6, 12, 18, 24, 30 and 36 months later.

### Bio-efficacy (Dalamba, village A)

Bio-efficacy tests will use the net samples from village A and will be conducted in laboratories. The knockdown, mortality and blood feeding tests will be conducted in Burkina Faso while those for egg production and production of viable larvae will be conducted at the Liverpool School of Tropical Medicine, UK.

### Mosquitoes

The knockdown and mortality tests will use a standard laboratory colony of permethrin-susceptible *An. gambiae* s.s. (Kisumu strain) aged 2 to 5 days old without blood feeding. The tests for egg and larvae production will use standardised laboratory colonies of permethrin-resistant *An. gambiae* s.s. (Tiassale strain) with blood feeding. This resistant colony will be submitted to regular quality testing, both phenotypic and genotypic, during the study to control for any changes in resistance levels.

### Test procedures

- Measuring knockdown and mortality

Knockdown and mortality will be measured using WHO cone bioassays [12]. Since 3 net samples will be cut from each net, there will be 72 netting samples to be tested for each arm at each time point and a total of 1,008 samples overall. Two standard WHO cones are fixed with a plastic manifold onto each of the three netting pieces, one at a time. Five permethrin-susceptible, non-blood-fed, 3- to 5-day-old female *An. gambiae s.s.* will be exposed for 3 minutes in each cone and then held for 24 hours with access to 6% sugar solution. Knockdown will be measured 60 minutes after exposure, and mortality measured after 24 hours. A negative control, from an untreated net, will be included in each round of cone bioassay testing. Bioassays will be carried out at 27 ± 2°C and 80 ± 10% relative humidity. Bioassay results for the netting pieces from each sampled net will be pooled to determine if the net meets the WHO efficacy requirement: that is ≥ 80% mortality. If the net fails these criteria, a tunnel test will be conducted on one of the four net samples that caused mortality closest to the average mortality in the cone bioassay [12].

b) Measuring egg production and viable offspring

Here the assay is designed to mimic what is likely to happen in nature by measuring egg and larval production in blood-fed, permethrin-resistant mosquitoes after exposure to the netting samples. Batches of 10, 3-day-old unfed mosquitoes will be exposed to LLIN, PPF-LLIN or untreated netting samples for 3 minutes using WHO cone bioassays [10]. At each net sampling time point, the same three panels will be tested for the six nets sampled from the field for each of the two types of netting. In addition a control untreated netting will be tested in duplicate. So for each of the 7 time points, 380 mosquitoes are tested (10 mosquitoes/test × 3 net panels/net × 6 nets/net type × 2 net types, plus 2 control nettings).

After exposure to the netting samples the female mosquitoes will be transferred to cages containing an equal number of newly emerged male mosquitoes and provided with 6% glucose solution *ad libitum*. Then, 24 hours after exposure to the netting samples the female mosquitoes will be fed on animal blood using membrane feeders for 25 minutes and any unfed mosquitoes recorded and discarded. After feeding, gravid mosquitoes will be individually placed into 15-cm^3^ cages, provided with an oviposition substrate of tap water in a 100 ml glass cup and left overnight to lay eggs. The following morning the number of eggs laid by individual females will be recorded. Eggs laid by each female will be transferred into individually-labelled plastic cup and the number of eggs hatching every 24 hours will be monitored for 3 days.

### Insecticide content (Dalamba, village A)

Insecticide content is the amount of active ingredient per gram of netting as determined by chemical assay. The net pieces of 30 cm^2^, cut from intact nets will be used to determine insecticide content. A sample from position 1 of the net will be included in baseline assays, but not in subsequent assays. Net samples will be weighed to estimate their density (mass of net per unit area) and then the samples from the same net will be combined for chemical analysis. The results will estimate the total active ingredient content of permethrin on the LLIN and pyriproxyfen and permethrin on PPF-LLIN. The Liverpool School of Tropical Medicine will conduct the chemical assay using analytical methods (Collaborative International Pesticides Analytical Council) recommended by WHO for quality control will be used to determine the total content of the active ingredient (unpublished method).

### Net survivorship (Sanako, village B)

To measure survivorship, all nets distributed to study subjects will be recorded. At each time point study nets will be visited and the physical presence of the net recorded. Those hanging over a bed will be considered as being in use. If the net is still present in the household, the investigator will record whether the net is being used for its intended purpose. Nets that have never been used will be recorded but excluded from the main analysis. If the net is no longer in the house, the investigator will determine how it was lost. This will be categorised as follows: loss by giving away the net to others to use either voluntarily (given to friends and relatives or sold) or involuntarily (stolen), and loss due to destruction, discarding or using it for alternative purposes because it is perceived as ‘useless’ [14]. These data will allow an estimation of the ‘hanging rate’ of bednets and allow us to better understand the reasons for net attrition.

### Fabric integrity (Sanako, village B)

Fabric integrity will be assessed from the questionnaire by counting the number of holes, including tears in the netting and split seams, by their location on the net and their size. Holes will be classified using the proportionate Hole Index (pHI) [14] into the following categories: good (pHI range 0 to 64), acceptable (pHI 65 to 642) and torn (≥643).

### Environmental measurements

The indoor temperature and relative humidity will be recorded continuously in 6 randomly-selected houses in each village from 0 to 36 months after net distribution to establish the environmental conditions present in the study houses. Data loggers (Lascar EL-USB-2-LCD Data Logger 80°C 100%RH) will be positioned at 1 metre above the floor on the wall nearest the foot end of the bed. Measurements will be made once an hour and the data will be downloaded every month.

### Measurement of adverse events

We consider that any AE arising from either net are highly unlikely. Nonetheless to document the safety of the use of these tools in a community setting, as in the main AvecNet Trial [9], residents will be followed for possible side effects of the PPF-LLIN by surveillance of known asthmatic subjects during the first month post-distribution and pregnancy outcomes will be monitored from antenatal clinic records.

### Safety considerations

There are no apparent risks to the safety of individuals or communities in this study. Permethrin-treated long-lasting nets have been fully evaluated by the WHO Pesticide Evaluation Scheme and approved for vector control (www.who.int/whopes/en/), and the products will be used in compliance with their recommended use and guidelines. The WHO hazard classification of permethrin is Class II, moderately hazardous [16]. The WHO hazard classification for pyriproxyfen is: U, unlikely to present acute hazard in normal use [16]. Pyriproxyfen is applied to drinking water sources for vector control because of its high insecticidal activity and low mammalian toxicity [3]. A recent WHO document [17] illustrates the low toxicity of pyriproxyfen for mammals: ‘*pyriproxyfen was not toxic when administered dermally to rats for 21 days at doses of up to 1,000 mg/kg of body weight per day. Inhalation of pyriproxyfen for 4 hours per day for 28 days caused only minor effects in rats (initial salivation, sporadically reduced body weight gain, slightly increased serum lactate dehydrogenase activity) at 10,000 mg/m*^*3’*^.

### Handling of drop-outs/withdrawals

If an individual wants to terminate his/her participation, no further follow-up will be performed. Net owners who have withdrawn consent, or are no longer in the study for whatever reason, will be replaced by other net owners resident in the same compound when possible. If a village opts out of the study during the first year, replacement by a neighbouring village will be considered.

### Study endpoints

#### Primary: bio-efficacy

Bio-efficacy is measured as percentage adult female mortality 24 hours after exposure to netting and as the percentage of viable offspring (first or second stage larvae) relative to the control group.

### Secondary: fabric integrity

Fabric integrity will be as defined earlier using the pHI [14] grouped into the following categories: good (pHI range 0 to 64), acceptable (pHI 65 to 642) and torn (≥643).

### Tertiary: net survival

Comparisons will be made between functional LLIN survival [14] with both types of nets.

### Tertiary: chemical analysis

The content of permethrin and pyriproxyfen in netting samples will be expressed in both g/kg and mg/m^2^.

### Sample size rationale

#### Primary outcome

##### Bio-efficacy - measured in Dalamba, village A

WHO expects that LLIN netting will kill at least 80% of mosquitoes that land on it. In this trial, however, the effects of the PPF-LLIN netting are not just on killing mosquitoes, but also causing sterility and reducing the number of offspring produced by female mosquitoes. A 50% reduction in killing when exposed to LLINs nets is likely to impact malaria vector control, as is a 50% decrease in the proportion of gravid females laying eggs after exposure to PPF-LLINs. In order to detect 50% reduction in killing from 80% to 40% and a coefficient of variation between clusters of 0.25, a conservative estimation, one requires 6 compounds with 4 nets, each with 3 netting samples/net/time, in each cluster to demonstrate a statistically significant impact at the 5% level and with 80% power. These calculations are based on the formula of Hayes and Bennett for clustered data [18]. For the 7 time points, one needs to recruit forty two compounds to each study arm.

#### Secondary outcome

##### Fabric integrity - measured in Sanako, village B

Since LLIN and PPF-LLIN are manufactured with the same fibre, the same weave and are manufactured by the same company we would not expect to find a difference in fabric integrity between them. However, a control programme manager might consider replacing all the nets in an area if > 25% were of poor condition. To estimate 25% nets in poor condition with a precision of ±10%, with a design effect of 1.0 because of cluster sampling, at the 5% level of significance, assuming there are either 500 LLINs or PPF-LLINs, would require 64 nets in each arm at the last sampling time. To allow for a loss of 10% of nets per year and for operational uniformity it is simplest to maintain the study framework of 100 nets in each group to allow for loss to follow-up if net users move away or stop using their nets; in which case, we would sample four nets in twenty-five compounds in each study arm. In addition, direct comparisons will be made between both nets.

#### Tertiary outcomes

##### Net survival - measured in Sanako, village B

For simplicity and to maximise precision of this measure, all nets will be sampled at 6-monthly time intervals: that is 374 of LLIN and 167 of PPF-LLIN. Direct comparisons of functional LLIN survival will be made between both net types using Kaplan-Meir analysis.

### Chemical analysis measured in Dalamba, village A

A 75% decline in the total content of either permethrin or PPF would cause concern since this is likely to impact the operational effectiveness of PPF-LLIN. The total concentration of PPF and permethrin in the fibres will be compared to the insecticidal activity overtime to examine their relationship. The manufacturers certify that the PPF-LLIN contain 2.0 g permethrin/g fibre, with range 1.5 g to 2.5 g and 1.0 g PPF g/g fibre, with range 0.75 to 1.25 g. In order to detect a 75% reduction in insecticide from 2.0 g to 0.5 g with a coefficient of variation of 0.25 (considered appropriate for factory-produced products where variation between nets is considered small) at the 5% level of significance and 80% power we will require 4 compounds in each study arm with 5 nets tested in each compound (n = 20 nets in each group). Since nets will be damaged during sampling they will be replaced with a new intact net. For this reason it is important that the date at which the net was put in place is recorded directly on each net.

### Data handling and record keeping

Entomological and environmental data will be recorded by field assistants and the clinical data by study nurses on standardised data forms. Each net will have a unique ID number and study subjects participating in the cross-sectional surveys will have their demographic surveillance number. All data recorded on individuals will be made by recording their demographic surveillance number, the village of residence and initials of the study subject; the initials will not be entered into the dataset to maintain anonymity.

Data forms will be double-entered, firstly by field staff and then by data entry staff, and the entries combined and errors corrected to produce a single dataset. This will be submitted to consistency checking by generic and study specific algorithms designed to identify sources of error. When inconsistencies occur, these will be checked against the original forms and subsequently amended in the dataset. Errors will be corrected when possible, with checking in the field when necessary and possible, to produce final datasets.

All forms with subject names and/or clinical data will be kept in a cabinet and locked when not in use; the key will be kept by the study coordinator or delegate. The electronic datasets will be password protected. Hard copies of the data will be stored for 10 years. The clinical data will be kept separately from that containing personal information. All forms and datasets, apart from those for enumeration, will identify subjects by their demographic surveillance number and initials and names will not be collected and initials will not be entered. After the collection and verification, data will be moved to data management unit in Banfora for data entry. The datasets will be password protected and only accessed by authorised study staff and the study data manager until after publication. Data will be stored for at least 10 years.

The PI will ensure that appropriate medical and research records for this study are maintained in compliance with Good Clinical Practice (GCP) and regulatory and institutional requirements. Authorised representatives of the sponsor, the ethics committee(s) or regulatory bodies may inspect all documents and records required to be maintained by the investigator. The PI, or designee, will ensure the access to facilities and the records.

### Data analytical plan

#### Bio-efficacy

##### Mortality

If the mortality in the control is between 5% and 20%, the data will be adjusted with Abbott’s formula shown below:

- Adjusted % mortality = (% alive in control - % alive in treatment) × % alive in control
- If the mortality in the control is > 20%, all the tests will be discarded for that day.
- General linear modelling will be used to assess the activity of the different insecticide treatments over time, adjusting for compound. Kaplan-Meier survival analysis will be used to compare treatments over time.

### Reduction in offspring

If less than 20% of gravid females in the control group fail to lay eggs the experiment will be repeated.

General linear modelling will be used to assess the activity of the different insecticide treatments over time, adjusting for compound. Kaplan-Meier survival analysis will be used to compare treatments over time.

### Fabric integrity

Three indicators will be calculated at each survey time: 1) the proportion of torn nets, 2) the proportion of nets with holes and 3) a hole index.

Proportion of nets in poor condition (with 95% confidence interval):

- Numerator: total number of each type of net where the nets are not long enough to be tucked under the mattress, or are torn or badly damaged, or have more than 5 holes (finger-width, approximate diameter 2 cm)
- Denominator: total number of each net type found and assessed in surveyed households

Proportion of nets with any holes (with 95% confidence interval):

- Numerator: fabric integrity will be defined using the pHI [14] grouped into the following categories: good (pHI range 0 to 64), acceptable (pHI 65 to 642) and torn (pHI ≥ 643)
- Denominator: total number of each net type found and assessed in surveyed households

The hole index would be calculated as:

Hole index = (0.5 to 1.9 cm^2^ × number of size-1 holes) +(2.0 to 9.9 cm^2^ × number of size-2 holes) + (10.0 to 24.9 cm^2^ × number of size-3 holes) + (≥25.0 cm^2^ × number. of size-4 holes)

The holes will be weighted according to the average area of each hole category. For the hole size categories described above, the weights would be 1, 23, 196 and 578, which corresponds with the areas estimated on the assumption that the hole sizes in each category are equal to the mid-points.

For each net type, the mean and standard deviation as well as the median and inter-quartile range of the hole index will be determined. Comparisons between treatments at different periods will be made using generalised linear models, adjusting for compound.

### Net survival

The analysis will include data on all nets recorded at each time interval. The number of nets in the sample, the proportion of the indicator and 95% confidence interval will be reported. The following indicators will be used and disaggregated by survey time (for example, 0 weeks, 6, 12, 18, 24, 30 or 36 months):

- Survivorship:
  Numerator: total number of nets in ‘good’ condition (pHI 0 to 64) plus ‘acceptable’ condition (pHI 65 to 642) hung over a bed × 100
- Denominator: total number of each net type distributed to surveyed households, excluding a portion (those that may be still in use by others) of the number lost by attrition

Attrition rate-1 for nets that have been destroyed or disposed of:

- Numerator: total number of each net product documented as lost due to wear and tear
- (‘torn’, pHI > 642) in surveyed households × 100
- Denominator: total number of each LLIN type distributed to surveyed households, excluding the number lost by attrition

Attrition rate-2 for nets not available for sleeping under:

- Numerator: total number of each net product reported as lost for reasons other than ‘torn’ (given away, stolen, sold or used in another location) in surveyed households × 100
- Denominator: total number of each net product distributed to surveyed households
- Attrition rate-3 for nets used for other purposes:
- Numerator: total number of each net product reported as being used for another purpose in surveyed households × 100
- Denominator: total number of each net product distributed to surveyed households
- For each net product, the survivorship rate plus attrition rate-1, attrition rate-2 and attrition rate-3 will add up to 100%.
- Kaplan-Meier survival analysis will be used to assess survivorship of nets with the log rank test being used to ascertain whether there are statistically significant differences between the two types of netting.

### Chemical analysis

The mean concentration and 95% confidence intervals of permethrin and pyriproxyfen will be measured on netting at time 0, and after 6, 12, 18, 24, 30 and 36 months.

### Environmental measurements

The maximum and minimum indoor temperature and relative humidity will be determined monthly for both villages during the study.

### Ethical approval

This study is conducted in accordance with the principles set forth in the ICH Harmonised Tripartite Guideline for GCP and the Declaration of Helsinki in its current version, whichever affords the greater protection to the participants. This protocol has been approved by the Ethics Committee for Research in Health, Ministry of Scientific Research and Innovation, Burkina Faso (2014-0-0250) and the School of Biological Sciences ethics committee, Durham University, UK (SBBS/EC/PV120914).

## Discussion

Insecticide-treated bednets are a highly effective vector control tool [19] but their future success is threatened by the spread of vectors resistant to pyrethroids [4], which is presently the only class of insecticide used for treating bednets. Finding alternative strategies to deal with the problem of insecticide resistance is a priority for malaria control and strategies leading to elimination [20]. Since bednets are effective methods of malaria control and have been used in their millions across SSA, insecticide resistance strategies that focus on bednets have much to recommend them. Here, we test a new net with a combination of two active ingredients: permethrin, a pyrethroid, and pyriproxyfen, a juvenile hormone mimic. Unlike permethrin, the principal mode of action of pyriproxyfen is not to kill mosquitoes, but to reduce their fecundity and fertility following a blood meal [6-8].

Ideally, the new combination must be effective for several years in order for it to be a useful public health intervention and to be recommended by WHOPES. In this trial we follow the recent recommendations made by WHOPES for assessing the durability of LLINs [12] and describe them in a protocol for others to adapt. Net durability will be assessed using a number of criteria. Specifically, this includes measuring the bio-efficacy and chemical content in the nets over time in one village, as well as the net survivorship and fabric integrity in a separate nearby village. Although we think it unlikely that there will be any adverse reactions to the new net containing pyriproxyfen we will record any AE in asthmatics and pregnant women.

The chemical measurement with time will give an indication of the longevity of the pyrethroid and pyriproxyfen in polyethylene netting in a typical rural village setting and the bioefficacy measures will give an indication of the activity of these compounds on the surface of the netting. The study is being run for 3 years as this is the current estimation of time between large-scale donation campaigns adopted by WHO and donor agencies, which balances expected net survival time and cost.

The nets used in the current study are of polyethylene, which was selected as the chemicals are incorporated into the fibres, which increases the duration for which pyrethroid is available and effective on the surface of the fibres. However, the duration of the pyriproxyfen is not known. The bio-efficacy measurements on pyriproxyfen include measurement of survival and fecundity of pyrethroid resistant *An. gambiae s.s.* after exposure to both the pyrethroid only net and that with pyriproxyfen over time. The effect of washing, in a village setting, on insecticide durability will also be examined.

If we can demonstrate in our main trial that large-scale use of the PPF-LLIN net reduces clinical malaria in children and in this trial that the nets are durable, remaining effective for a number of years in an area with pyrethroid-resistant vectors, then the results of these trials will be of interest to malaria control programmes in Burkina Faso and other parts of SSA.

## Trial status

Recruiting.

## Additional file

Additional file 1:
**Partie 1 Formulaire d’information pour les réunions communautaires.**

## Abbreviations

AE: adverse events
GCP: Good Clinical Practice
CRF: case report form
GPS: global positioning system
ICH: International Conference on Harmonisation of technical requirements for registration of pharmaceuticals for human use
ID: identity number
LLIN: long-lasting impregnated nets (permethrin-treated)
pHI: proportionate Hole Index
PPF-LLIN: permethrin and pyriproxyfen long-lasting impregnated nets
PI: Principal Investigator
SSA: sub-Saharan Africa
WHO: World Health Organisation
WHOPES: WHO Pesticide Evaluation Scheme
