# Supplementary material for: To assess whether addition of pyriproxyfen to long-lasting insecticidal mosquito nets increases their durability compared to standard long-lasting insecticidal mosquito nets: study protocol for a randomised controlled trial
Source: Trials. 2015 Apr 28;16:195. doi: 10.1186/s13063-015-0700-7 (PMC4423132; doi:10.1186/s13063-015-0700-7)
Supplement: Additional file 1: — Partie 1 Formulaire d’information pour les réunions communautaires. [file 13063_2015_700_MOESM1_ESM.docx]

**Annexe 1
*Partie 1 Formulaire d’information pour les réunions communautaires***

Protocole : Version 1.0 Du 19 Février 2014

Evaluation de l’effet additionnel du pyriproxyfène sur la longévité des moustiquaires imprégnées d'insecticide à longue durée d’action comparées aux moustiquaires imprégnées d'insecticide à longue durée . Protocole pour une étude randomisée en grappe.

Information à communiquer par les enquêteurs, superviseurs ou infirmiers (es).

Veuillez traduire ce qui suit dans la langue locale appropriée à la communauté réunie. (Dire la prière selon les coutumes).

Bonjour/Bon après midi/Bonsoir, merci d’être présent à cette rencontre. Comme vous le savez déjà, le CNRFP travaille dans la recherche sur le paludisme.

Comme vous le savez probablement, le paludisme est une maladie importante au Burkina Faso et se transmet d'une personne à une autre par piqûre de certains moustiques. Ces moustiques piquent généralement la nuit. Dormir sous une moustiquaire protège contre les moustiques qui piquent pendant la nuit. Si la moustiquaire a été traitée avec un produit chimique qui tue les insectes (insecticides), il donne une meilleure protection contre les piqûres de moustiques. Certains types de moustiquaires reçoivent un traitement spécial à l'usine et ne nécessitent pas de retraitement jusqu'à la fin de leur vie ; ceux-ci sont appelés moustiquaires imprégnées d'insecticide à longue durée d’action (MILDA).

Nous distribuerons deux types de MILDA pour la prévention et le contrôle du paludisme dans les zones ciblées pour cette intervention. La communauté dans laquelle vous vivez a été ciblée pour recevoir les deux types de moustiquaires. Nous voulons mesurer combien de temps chaque type de moustiquaire dure pour une utilisation courante dans les ménages de votre communauté. Votre village a été sélectionné pour cette étude. Des moustiquaires vont être distribuées, telles que chaque ménage aura au moins une moustiquaire sous laquelle dormir. Notre équipe va ensuite venir dans votre village, pour déterminer pendant combien de mois ces moustiquaires ont été utilisées au-dessus de lits et pendant combien de temps les produits chimiques restent sur ces moustiquaires imprégnées.

Que se passera t-il?: Si vous acceptez de participer, la procédure suivante sera suivie:

1. Nous allons faire un recensement dans le village afin de déterminer le nombre de

moustiquaires et les différents types de concessions.

2. Au début de l’étude, chacun aura une moustiquaire imprégnée d’insecticide sous laquelle il dormira au début de l’étude en Mai 2014. Cette moustiquaire contiendra uniquement un seul type d’insecticide. Vos anciennes moustiquaires seront retirées contre de nouvelles et pour ceux qui n’en possédaient pas, il leur sera donné une nouvelle moustiquaire. Il vous sera offert une moustiquaire pour deux et nous vous aiderons à les placer au lieu où vous dormez. On vous demandera de dormir sous ces moustiquaires chaque nuit. Pensez-vous qu’il vous sera difficile de le faire ?

3. Lors de nos passages nous aimerions prendre quelques moustiquaires avec nous, et mesurer quelle quantité d’insecticide est encore là et quelle quantité est parti. Si votre moustiquaire a été sélectionnée et vous êtes d’accord de nous donner la permission de prendre cette moustiquaire, nous allons immédiatement vous en donner une de remplacement, que vous pourriez attacher au-dessus de votre lit. Pensez-vous qu’il vous sera difficile de le faire ?

4. Avec votre accord, nous passerons au village deux fois dans l’année après la distribution des moustiquaires pendant trois ans, pour vérifier dans quelles conditions les moustiquaires sont utilisées.

5. Ces moustiquaires sont sans dangers mais les, nouvelles moustiquaires imprégnées par les deux insecticides n’ont pas encore été utilisées par un grand nombre de personnes comme les moustiquaires imprégnées avec un seul insecticide. De ce fait, nous vous poserons des questions sur votre appréciation de ces nouvelles moustiquaires. Nous vérifierons aussi s’il y’a des personnes soufrant d’asthme dans le village, et comment ils apprécient les nouvelles moustiquaires. Nous vérifierons aussi que les nouvelles moustiquaires ne causent pas de problèmes aux femmes enceintes et à leurs bébés.

6. Nous voulons vérifier l’efficacité à tuer les moustiques de ces moustiquaires dans le temps. Pour cela les moustiquaires que nous allons récupérer avec vous seront testées en mettant en contact les moustiques avec ces moustiquaires et vérifier leur effet.

Risques: Il existe plusieurs informations qui prouvent que les insecticides utilisés sur les

moustiquaires sont uniquement nocives aux moustiques et aux insectes. Cependant, on vous demandera si vous avez déjà eu un problème lié à l’utilisation des moustiquaires.

Bénéfices: Chaque lit/lieu pour dormir sera gratuitement doté d’une moustiquaire imprégnée d’insecticide à longue durée.

Vous êtes libre de quitter l’étude à tout moment. Pour ce faire, vous pourrez oralement informer un membre de l’équipe du CNRFP ou par écrit sans aucun préjudice mais nous ne pourrions pas vous remettre l’ancienne moustiquaire.

Merci d’être venu à cette rencontre. Nous restons disponibles pour toute question qu’il vous plairait de poser.

Fin de la rencontre (Dire la prière selon les coutumes)*.*

***Partie 2. Formulaire d'information pour les ménages sélectionnés***
**Introduction**

Je m’appelle <……………..>, et j’ai avec moi <……….…..> et nous travaillons pour le Centre National de Recherches et Formation sur le Paludisme. Je vous invite à lire ou écouter attentivement ce que nous vous expliquons dans une langue que vous comprenez bien, avant d’accepter de participer à l’étude. Le but de cette étude est de vérifier l’action d’un nouveau type de moustiquaires traitées avec un insecticide. Nous nous attendons à ce que les moustiquaires gardent leur capacité à tuer les moustiques vecteurs du paludisme après plusieurs lavages et 3 ans ou plus d’usage. Cette étude a été approuvée par le comité d’éthique du Burkina Faso.

**Objet et contexte de l’étude**

Tout d'abord quelques informations de base. Comme vous le savez probablement, le paludisme est une maladie importante au Burkina Faso et se transmet d'une personne à une autre par piqûre de certains moustiques. Ces moustiques piquent généralement la nuit. Dormir sous une moustiquaire protège contre les moustiques qui piquent pendant la nuit. Si la moustiquaire a été traitée avec un produit chimique qui tue les insectes (insecticides), il donne une meilleure protection contre les piqûres de moustiques. Certains types de moustiquaires reçoivent un traitement spécial à l'usine et ne nécessitent pas de retraitement jusqu'à la fin de leur vie ; ceux-ci sont appelés Moustiquaires imprégnées d'insecticide à longue durée d’action (MILDA).

Nous distribuerons deux types de MILDA pour la prévention et le contrôle du paludisme dans les zones ciblées pour cette intervention. La communauté dans laquelle vous vivez a été ciblée pour recevoir les deux types de moustiquaires. Nous voulons mesurer combien de temps chaque type de moustiquaire dure pour une utilisation courante dans les ménages de votre communauté. Votre village a été sélectionné pour cette étude. Comme vous le savez peut-être, nous avons demandé à vos dirigeants la permission d’effectuer cette étude. Ensuite, les moustiquaires vont être distribuées, telles que chaque ménage aura au moins une moustiquaire sous laquelle dormir. Mon équipe va ensuite venir dans votre village, en accord avec les chefs de votre communauté ou village, pour déterminer pendant combien de mois ces moustiquaires ont été utilisées au-dessus de lits et pendant combien de temps les produits chimiques durent.

***Informations sur les moustiquaires de l'étude***

Les moustiquaires de l’étude que nous allons donner à votre ménage ont été traitées à l’usine. Dans cette étude, les produits chimiques utilisés sont : la perméthrine seule ou la perméthrine et le pyriproxyfène combinés. Notez que ces produits ne sont pas nouveaux : ils sont bien établis, mais n’ont jamais été combinés dans une moustiquaire auparavant. Nous conduisons actuellement une étude dans d’autres villages proches du votre pour vérifier si les moustiquaires avec les deux produits chimiques sont plus efficaces pour réduire le paludisme que celles avec un seul produit. La question que nous nous posons dans cette étude est « Combien de temps durent ces moustiquaires ? ».

**Type d’étude**

Nous allons fournir de nouvelles moustiquaires à votre ménage, et vous demander de les suspendre au-dessus de vos lits/places de couchage. Nous allons prendre les moustiquaires que vous avez actuellement pour éviter une confusion. Nous allons ensuite suivre les moustiquaires au fil du temps, pour voir à quelle vitesse elles se trouent et s’usent, et combien de produit chimique elles ont encore, en choisissant quelques moustiquaires pour collecter les informations dans 6 mois et ensuite après 12, 18, 24, 30 et 36 mois. Le choix des moustiquaires pour collecter les données sera fait au hasard. Si nous prenons votre moustiquaire pour mesurer les produits chimiques, vous en recevrez une nouvelle. Un plus petit nombre des ménages, choisis au hasard, sera revisité chaque mois pour voir si les moustiquaires sont toujours utilisées et encore en bon état.

***Sélection des participants***

Après que cette région ait été sélectionnée pour l’étude, nous avons donné un numéro à chaque ménage. Pour choisir les ménages pour les visites de suivi, nous avons utilisé un ordinateur qui choisit des nombres comme à la loterie, et c’est comme ça que nous avons choisi les moustiquaires à suivre. Nous vous demandons un entretien car votre moustiquaire fait partie des moustiquaires sélectionnées, ou parce que vous êtes le parent/gardien/chef de ménage d’un enfant qui utilise une de ces moustiquaires.

***Procédures***Je voudrais donc avoir votre consentement à être interviewé ; ceci va prendre environ <temps approximatif> minutes. Pendant l’entretien, je vais vous poser quelques questions sur votre ménage, le statut de la moustiquaire que vous avez reçu ou votre enfant a reçu et comment vous l’utilisez et la manipulez. Je vais vous demander de me montrer la moustiquaire pour que je puisse voir comment elle est utilisée.

<NOTE à l’interviewer : Si la moustiquaire a été sélectionnée pour le bio-essai, lire « A » ci-dessous ; si la moustiquaire n’a pas été sélectionnée pour le bio-essai, lire « B ».>

A – Nous aimerions prendre quelques moustiquaires avec nous, et mesurer quelle quantité d’insecticide est encore là et quelle quantité est parti. Votre moustiquaire a été sélectionnée par hasard pour ce test. Si vous êtes d’accord de nous donner la permission de prendre cette moustiquaire, nous allons immédiatement vous en donner une de remplacement, qui va être la vôtre dès aujourd’hui et que vous pourrez suspendre au-dessus de votre lit.

B – Nous n’allons pas endommager votre moustiquaire, après cet entretien, nous allons vous la redonner. A la fin de l’étude, tous les villageois seront informés des principaux résultats de l’étude lors d’une réunion de la communauté dans le village.

***Confidentialité***Toutes les informations relatives à votre participation seront gardées confidentielles et ne vont pas être transmises, sauf si requises par la loi, comme pour une demande légale de la liste des bénéficiaires. Votre identité ne sera révélée dans aucun rapport ou publication résultant de l’étude. Les résultats de l’entretien seront enregistrés dans un ordinateur, mais avec les codes des ménages et non pas les noms des personnes interviewées. Les données collectées seront gardées pour l’analyse. Elles seront gardées pour quelques temps sur papier et ordinateur, mais seront finalement détruites au bout d’un temps défini par l’étude.

***Participation volontaire : droit de refuser ou de retirer son consentement***Votre participation aux entretiens est entièrement volontaire. Vous n’êtes pas dans l’obligation de participer et vous avez le droit de refuser cette invitation. Si à tout moment de l’entretien vous ne voulez plus participer, vous êtes libre de vous retirer immédiatement, sans discussion supplémentaire ; ceci n’aura pas de conséquences adverses pour vous. Que vous décidiez de participer au pas, vous allez recevoir les services publics que vous recevez habituellement. Les moustiquaires de l’étude qui vous ont été données vous appartiennent et vous pouvez les garder. Dans certains cas, nous allons peut-être vous demander de nous donner une ancienne moustiquaire en échange d’une nouvelle de remplacement, mais vous pouvez refuser cette demande si vous le souhaitez.

**Qui contacter ?**

Si vous avez des questions, n’hésitez pas à les poser maintenant. Si vous désirez poser des questions plus tard, vous pouvez contacter une des personnes suivantes :

Dr Sagnon N’Falé au numéro de téléphone suivant 70 23 91 09 ou Dr Moussa Guelbeogo, au numéro de téléphone suivant 70 33 51 46. CNRFP, 01 BP 2208 Ouagadougou 01, Burkina Faso. Téléphone : 50 32 46 95

Toute nouvelle information importante concernant les résultats de notre étude vous seront transmises.

Ce projet a été revu et approuvé par le comité d’éthique du Burkina Faso, dont la tache principale est de s’assurer que les participants à l’étude sont protégés de tout mal. Si vous voulez en savoir plus sur ce comité, vous pouvez contacter <nom, addresse et téléphone numero >.

Nous vous laissons une copie de ce consentement éclairé pour votre information et comme référence future.

***Partie 3. Fiche de consentement***

J’ai lu ces informations en français ou elles m’ont été lues dans ma langue maternelle. J’ai eu l’occasion de poser des questions sur le sujet, et on a répondu de manière satisfaisante à toutes mes questions. Je consens volontairement à participer à cette étude, et je comprends que j’ai le droit de me retirer de l’étude en tout temps sans que cela affecte mes droits. Je comprends aussi que l’Investigateur principal de l’étude peut exclure mon ménage de l’étude sans mon consentement. J’ai reçu une copie de cette fiche de consentement.

Imprimer le nom du participant :

Date et Signature du participant :

______________________________________ / ___ / ___ ( jj/mm/aa)

***Si analphabète***

J’ai été témoin de la lecture de la fiche de consentement au participant potentiel. La lecture a été soigneuse et précise et la personne a eu l’occasion de poser des questions. Je confirme que la personne a donné un consentement de manière libre.

Imprimer le nom du témoin lettré indépendant :

__________________________________
(Si possible, cette personne sera choisie par le participant et n'aura pas de lien pas de lien avec l’équipe de recherche)

Date et Signature du témoin

______________________________________ / ___ / ___ ( jj/mm/aa)
J’ai lu ou ai été témoin de la lecture de la fiche de consentement au participant potentiel. La lecture a été soigneuse et précise et la personne a eu l’occasion de poser des questions. Je confirme que la personne a donné un consentement de manière libre.

Imprimer le nom du chercheur :

_____________________________________________
Date et signature du chercheur

______________________________________ / ___ / ___ ( jj/mm/aa)
